# Comparative Therapeutic Validation of a Microfluidic Metastasis Model: Cimetidine but Not Cilengitide Inhibits Physiological Tumor Cell Extravasation

**DOI:** 10.3390/bioengineering13101192

**Published:** 2026-10-14

**Authors:** Claudia Kühlbach, Lea Olschewski, Frank Baganz, Volker C. Hass, Margareta M. Mueller

**Affiliations:** 1 Department of Biochemical Engineering, UCL University College London, London WC1E 6BT, UK; claudia.kuehlbach@hs-furtwangen.de (C.K.); f.baganz@ucl.ac.uk (F.B.); volkerclaus.hass@hs-furtwangen.de (V.C.H.); 2 Department of Health, Medical and Life Sciences, HFU Hochschule Furtwangen, 78054 Villingen-Schwenningen, Germany; lea.olschewski@hs-furtwangen.de

**Keywords:** metastasis, tumor cell extravasation, Cilengitide, Cimetidine, microfluidic device, HPAEC

## Abstract

Interactions between tumor cells and the endothelial lining of blood vessels under flow conditions are essential for successful metastasis. These interactions are mainly facilitated by adhesive proteins, like integrins or selectins and by proteases. While expression of specific integrins (α_v_β_3_ and α_v_β_5_) and selectins correlate with tumor progression in cancer and seem to mediate tumor cell adhesion, analysis of a therapeutic intervention in these processes is frequently limited to systems lacking physiological cell–cell interactions. In this study on the adhesion of different tumor cell types in monoculture and in coculture on endothelial cell layers was evaluated under different conditions, i.e., different static setups and under flow conditions in a microfluidic device and under treatment with the integrin inhibitor Cilengitide and the selectin inhibitor Cimetidine. This careful comparative experimental validation revealed that while Cilengitide inhibits H838-, HCT116- and U87-tumor cell adhesion in tumor cell monocultures in all assay set-ups its effect is abolished by the presence of endothelial cells. In contrast, Cimetidine, a Histamine 2 receptor antagonist and E-selectin inhibitor significantly reduced H838 lung carcinoma cell adhesion and trans-endothelial migration suggesting a significant role of E-selectin in tumor progressions and metastasis.

## 1. Introduction

Cell–cell interactions between tumor cells and vascular endothelial cells, i.e., tumor cell adhesion and transmigration und the physiological flow conditions of the vascular system are essential for the process of metastasis to distant organs [1,2,3] and are frequently mediated by integrins, selectins and proteases [2,3]. During adhesion of circulating tumor cells to the endothelium, one of the first crucial steps of the metastatic process [2], adhesive proteins, like integrins, selectins and members of the immunoglobulin family are known to play a central role [3,4,5,6,7,8,9]. Inhibiting the tumor–endothelial cell interaction, e.g., by inhibition of integrin function was thus considered a therapeutic target for diminishing tumor progression and metastatic dissemination [4,7,8,10,11,12].

Integrins are hetero-dimeric transmembrane proteins consisting of α- and β-chains [3,4,7,8,10,12]. They function as mechanosensors, adhesion molecules and signal transduction proteins in many physiological but also in pathological processes, making them an important target for the treatment of different diseases [10,13,14]. Tumor cell expression of integrins α_v_β_3_, α_v_β_5_, α_5_β_1_, α_6_β_4_, and of the α_4_ integrin subunit correlates with tumor progression in different cancer entities [3,4,15,16,17,18]. The integrin α_v_β_3_ was described to facilitate tumor cell arrest on blood vessel endothelial cells [1,3,10] and both α_v_β_3_ and α_v_β_5_ integrins are considered responsible for tumor cell adhesion, migration, proliferation and metastasis [11,14,16,19]. Activation of α_v_β_3_ and α_v_β_5_ integrins can be selectively blocked by Cilengitide a cyclic RGD pentapeptide (EMD 121974; Merck KgaA, Darmstadt, Germany) the clinically most advanced integrin inhibitor used in anticancer therapy [14,19].

Cilengitide inhibits cell adhesion and increases apoptosis after detachment of cells in vitro [7,14,19,20]. Indeed, inhibition of both α_v_β_3_ and α_v_β_5_ integrin resulted in blockade of cell adhesion up to 90% [2,4]. Accordingly, Cilengitide was previously shown to inhibit migration and invasiveness of melanoma and meningioma cells in vitro [18,21]. Additionally, inhibition of α_v_ integrin by Cilengitide treatment in cultures of brain tumor cells (DAOY (medulloblastoma), U87 and SF763 (glioblastoma)) and brain capillary endothelial cells resulted in cell detachment and apoptosis for both cell types in [22] an effect that was similarly observed in monocultures of glioma cells and different breast cancer cell lines [23,24]. Chilla et al. demonstrated a concentration-dependent decrease in cell attachment for breast cancer cells in vitro with increasing Cilengitide concentrations [11], an effect that was also observed upon Cilengitide treatment of osteosarcoma cell monocultures [25]. In addition, a Cilengitide-induced inhibition of cell migration, invasion and proliferation in vitro was observed in breast cancer cell lines [26], malignant pleural mesothelioma cells [27] prostate cancer, melanomas and gliomas [10].

In xenograft tumor models in vivo inhibition of α_v_ integrin resulted in a reduction in tumor growth and metastasis formation [3,7,10,28,29], an effect that might well be mediated by the inhibition of angiogenesis that was also observed in vivo. In contradiction to these results, the loss of integrin expression in α_v_ knockout mice increased tumor angiogenesis [30].

The anti-tumor activity of Cilengitide has been confirmed in several animal models [4,21,25,26,28]. In an in vivo study with hamsters, Cilengitide prevented melanoma metastasis formation [21,28]. Cilengitide also reduced metastasis volume in bone metastasis of breast carcinoma in animal models in vivo [26]. Treatment resulted in suppression of metastatic growth but exerted only minimal effects on the growth of the primary tumor [25]. In contrast, in a rat model of colon carcinoma, blockage of β_1_-, β_4_, and α_v_-integrins did not impair tumor cell adhesion in the lung [9].

Interestingly, Cilengitide exhibited additional effects beyond the inhibition of cell adhesion. Cilengitide treatment of glioblastoma cell cultures isolated from patients resulted in an enhanced growth inhibition when compared to temozolomide treatment. Cell lines also proved more sensitive to Cilengitide than to temozolomide when they had a specific methylated O6-Methylguanine-Methyltransferase promotor [31]. However, the effect was not very strong in a phase II clinical trial in glioblastoma [32]. Cilengitide in combination with temozolomide lead to a synergistic reduction in proliferation and tumor growth on melanoma cells both in vitro and in vivo [33]. These anti-tumor effects of Cilengitide could not be confirmed in a phase III clinical trial, where patients showed no benefit and therefore no better outcome from the additional treatment with Cilengitide as compared to temozolomide alone [34,35].

Surprisingly, analysis of the mechanisms underlying the anti-tumor effects of Cilengitide revealed induction of tumor necrosis and vascular disruption after systemic treatment with Cilengitide in animal models [23,36]. In agreement with these data vascular impairment could also been observed in vitro in endothelial cell cultures, where the addition of Cilengitide lead to reduced cell adhesion, increased cell detachment and a disruption of vascular endothelial (VE)-cadherin at adhesion junctions [37,38].

Taken together, data on the efficacy of Cilengitide as anti-tumor or anti-metastatic agent are somewhat controversial. Depending on the experimental set up, in vitro or in vivo analyses or clinical trials different efficacy profiles or lack thereof have been observed and the overall mechanism of action seems still somewhat elusive. Nonetheless, many publications, suggest that the integrins α_v_β_3_ and α_v_β_5_ influence the adhesion of tumor cells during the extravasation process, suggesting Cilengitide as inhibitor of an important step in the metastatic process.

Besides integrins another class of adhesion molecules, the selectins, play a role in endothelial cell interactions. Selectins are known to be involved in leukocyte adhesion and rolling and therefore in the initiation of the extravasation process [6,39]. They have been shown to play a role in leukocyte and tumor cell adhesion and migration as well as extravasation [2].

Cimetidine [N″-cyano-N-methyl-N’-(2(((5-methyl-1H-imidazol-4-yl)methyl)thio)ethyl) guanidine], is a Histamine 2- (H2-) receptor antagonist that is registered as treatment for gastric ulcer diseases and gastro esophageal reflux both of which are stimulated by histamine [40,41]. In addition, Cimetidine also acts as E-selectin inhibitor and has been described to have inhibitory effects on tumor cell proliferation, adhesion, invasion, and angiogenesis in different tumor entities [41,42]. There are studies demonstrating the inhibition of gastric and breast cancer cell adhesion on HUVEC endothelial cells in vitro by Cimetidine treatment [41,43,44] as well as an inhibitory effect of Cimetidine on the expression of E-selectin and thus on cell adhesion [41,42]. Indeed, treatment with Cimetidine results in an increased survival rate in animal models and cancer patients [45,46]. This might be the result of a dual effect of Cimetidine. Histamine is known to act as receptor-independent growth factor in some tumor malignancies, like breast or gastric cancer and melanoma cells and promotes proliferation. Consequently, Cimetidine can not only exhibit its effect as E-selectin inhibitor but also as antagonist of the H2-receptor [40,44].

To determine the potential contribution of integrins, inhibited by Cilengitide and of E-selectin, inhibited by Cimetidine, to the extravasation process as the first step in the metastatic cascade, Boyden chamber assays as well as extravasation experiments in an endothelial cell lined microfluidic device were performed, both under static as well as dynamic conditions.

## 2. Material and Methods

### 2.1. Cells and Culture Media, Antibodies and Reagents

Cells were cultured under standard conditions at 37 °C and 5% CO_2_. Human pulmonary arterial endothelial cells (HPAEC) and green fluorescent protein (GFP) transfected H838 non-small lung carcinoma cells (H838GFP) were cultured as described previously [47].

GFP transfected colon carcinoma cells HCT116GFP were cultured in Minimal Essential Medium alpha (MEMα), containing 10% fetal calf serum (FCS), 1% penicillin/streptomycin and 200 µg/mL G418. The cells were passed by incubation with 0.05% EDTA for 10 min, followed by 0.1% Trypsin/0.05% EDTA mix (1:1) for 3 min. GFP transfected glioblastoma cells U87GFP were cultivated in Roswell Park Memorial Institute Medium (RPMI-1640), containing 10% FCS, 1% penicillin/streptomycin and 200 µg/mL zeocin. The cells were passaged by incubation with 0.05% EDTA for 30 s, and subsequent incubation with 0.1% Trypsin/0.05% EDTA mix (1:1) for 2 min.

Cilengitide (EMD 121974, Sigma-Aldrich, Taufkirchen, Germany) was used as integrin α_v_β_3_, and α_v_β_5_ inhibitor.

Cimetidine (H2-Blocker, Ratiopharm, Ulm, Germany) was used as inhibitor of E-selectin.

### 2.2. Adhesion Assay

Adhesion assays were performed in 96-well plates with Cilengitide concentration of 17, 34, 68, 170 nM, and 340 µM, an α_4_ integrin inhibitory antibody (Bio-Techne, Wiesbaden, Germany) at a concentration of 2.5, 5 and 10 µg/ml and Cimetidine concentrations of 0, 10, 100, and 200 µM.

For monoculture assays, singularized H838GFP, HCT116GFP and U87GFP cells were incubated with Cilengitide, the α_4_ integrin antibody or Cimetidine at different concentrations for 10 min at room temperature and subsequently control cells and treated samples were seeded in triplets (4 × 10^4^ cells per well in 200 µL medium). The assay was incubated for 1 h under standard conditions, washed once with phosphate-buffered saline (PBS), fixed for 30 min with 80% ethanol and stained with hemalaun sauer solution (Carl Roth, Karlsruhe, Germany) for 180 min. Cells adherent to the culture plate were photographed. Total area of adherent cells was measured and evaluated, using Image J and Excel.

For coculture assays, 3 × 10^5^ HPAECs in 200 µL medium per well were seeded in triplets and incubated for 24 h under standard conditions. After 24 h, singularized H838GFP, HCT116GFP, or U87GFP cells were incubated with Cilengitide, the α_4_ integrin antibody or Cimetidine at different concentrations for 10 min at room temperature. Control and treated cells were then seeded in triplets (4 × 10^4^ cells per well in 200 µL medium) and incubated for 1 h under standard conditions. Subsequently, cells were washed once with PBS, fixed for 20 min with 4% of paraformaldehyde (PFA), followed by 70% and 100% ethanol, 5 min each. Cells were photographed and quantification of cell adhesion was achieved by counting the adherent GFP positive cells, using Photoshop CS6 and comparative evaluation of the results in Excel. Error bars indicate standard deviation (SD).

### 2.3. Treatment of Tumor Cells with Cilengitide in a Boyden Chamber

For monoculture assays, singularized H838GFP, HCT116GFP, or U87GFP were incubated with 340 µM Cilengitide for 10 min at room temperature and 7 × 10^3^ cells in 500 µL medium were seeded into the filter insert (the upper compartment) of a 24-well Boyden Chamber, with pore size 5 µm. The lower compartment contained 1.5 mL of medium. The assay was incubated for 1 h under standard conditions. Filter inserts were then washed once with PBS, fixed with 80% ethanol for 30 min and adherent cells were stained with hemalaun sauer solution for 180 min and photographed. Total area of cells was measured, using the free software Image J (version 1.54p) and evaluated with Excel. Error bars indicate standard deviation (SD).

For coculture assays, HPAECs were seeded in duplicates in different positions in the Boyden Chamber, either in the upper compartment, on the bottom side of the filter insert membrane or in the lower compartment. Alternatively, only HPAEC conditioned medium was used. Cell numbers seeded were 3 × 10^4^ cells in upper compartment or on the bottom side of the membrane, or 4 × 10^5^ cells in the lower compartment. 800 µL medium were added in the lower compartment. Cells were incubated for 24 h under standard conditions. After 24 h, singularized H838GFP, HCT116GFP, or U87GFP cells were incubated with Cilengitide 340 µM, for 10 min at room temperature, then seeded at a cell number of 4 × 10^4^ cells per well in 200 µL medium into the upper compartment of the Boyden Chambers and incubated under standard conditions for 1 h. The insert membranes were subsequently washed once with PBS and fixed with 4% PFA, 70% ethanol and 100% ethanol, 20 min, 5 min and 5 min, respectively. For quantification of adherent cells on the membranes GFP positive cells were counted, using Photoshop CS6 (version 25.12.2). Error bars indicate standard deviation (SD).

### 2.4. Treatment of Tumor Cells with Cimetidine in a Boyden Chamber

First, 3 × 10^4^ HPAECs were seeded in duplicates on the membrane in the upper compartment of the Boyden Chamber and incubated for 24 h under standard conditions. The following day, singularized H838GFP were incubated with 200 µM Cimetidine for 10 min at room temperature. Thereafter, 4 × 10^4^ tumor cells per well were seeded in 200 µL medium on top of the endothelial cells and incubated under standard conditions. After 1 h, the insert membranes were washed once with PBS and fixed with 4% PFA, 70% ethanol and 100% ethanol, 20 min, 5 min and 5 min, respectively. For quantification of adherent cells on the membranes GFP positive cells were counted, using Photoshop CS6. Error bars indicate standard deviation (SD).

### 2.5. Adhesion Assays in Microfluidic Device

The microfluidic device was assembled as described before [47]. For monoculture assays, the membrane sandwiched between the two channels was left without cells, for coculture assays, the entire upper channel in the device was seeded with HPAEC endothelial cells as described before [47]. Singularized H838GFP, HCT116GFP, or U87GFP were incubated with Cilengitide, 340 µM, or Cimetidine, 200 µM, for 10 min at room temperature. A total of 1 × 10^6^ tumor cells per ml were injected into the vessel equivalent of the microfluidic device and incubated without flow for 1 h under standard conditions. After incubation time, the device was taken apart. The membrane was washed once with PBS and fixed with 4% PFA for 20 min and with 70% ethanol and 100% ethanol, 5 min each.

For experiments under flow conditions, H838GFP lung carcinoma cells were singularized and incubated with Cilengitide or Cimetidine as described above. The cell suspension (1 × 10^6^ tumor cells per ml) was continuously introduced into the microfluidic device in media containing the respective drug treatment, using a microliter pump (Cetoni, Korbussen, Germany) with a flow speed of 8 mm/s, as this best represents the flow velocity in small arterioles, ranging from two to 20 mm/s [48]. This flow velocity corresponds to a shear stress of T = 4.6 dyn/cm^2^ (calculation see Supplementary Materials File SM1). The flow was maintained for 24 h. Subsequently, the device was taken apart and the membrane treated as described before. Quantification of the adherent cells was achieved by counting the GFP positive cells using Photoshop CS6. Error bars indicate standard deviation (SD).

### 2.6. Statistical Analysis

Statistics was performed by *t*-test analysis. Significance was defined as *p*-value of *p* = 0.05.

## 3. Results

**Figure 1:**
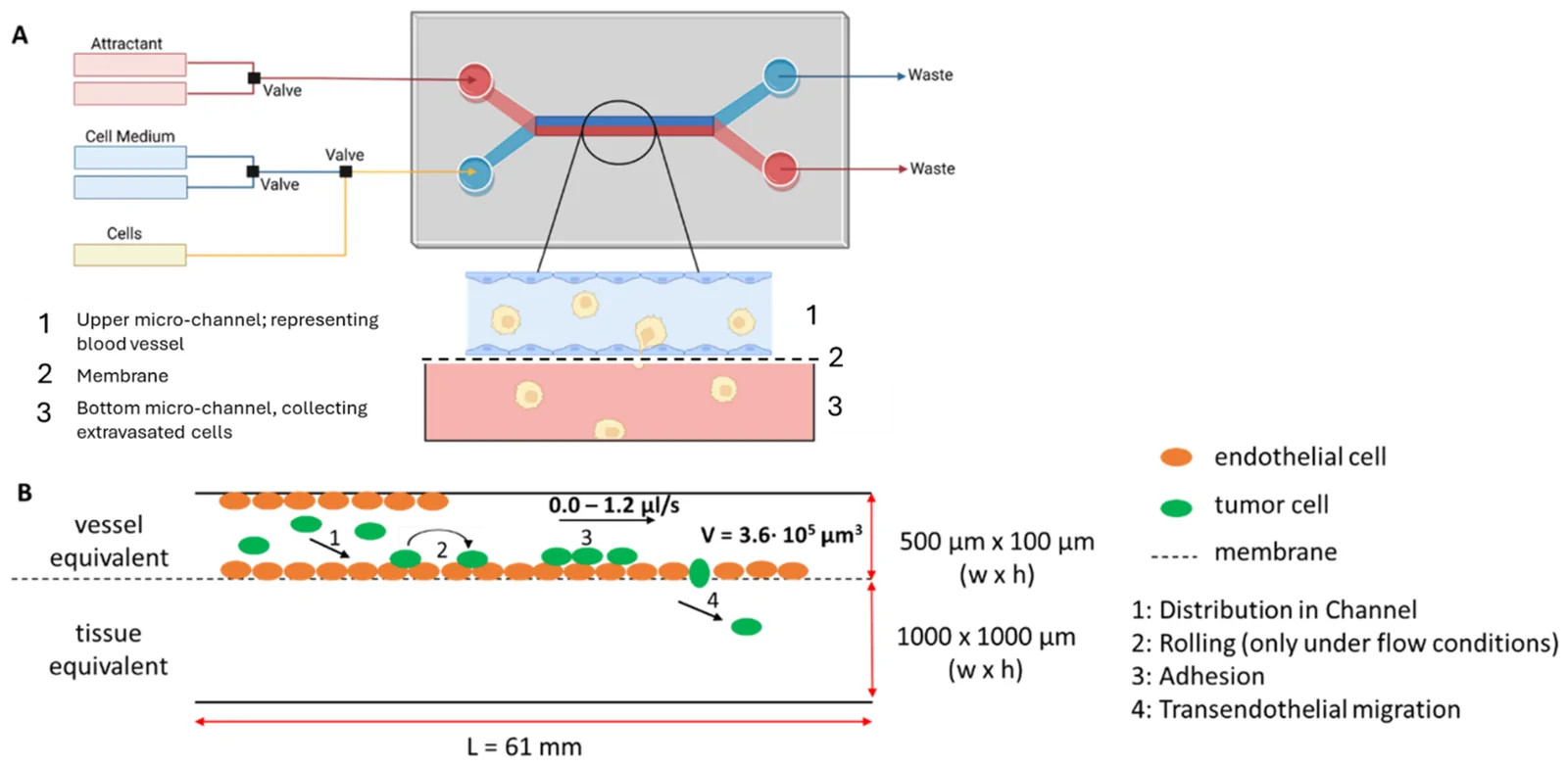
Schematic representation of the microfluidic device (**A**) and the extravasations steps within (**B**). (**A**): The microfluidic device consists of two parallel channels with a porous membrane sandwiched in between. Upper channel with membrane represents the blood vessel (upper channel dimensions 500 μm × 100 μm × 6.1 cm (width × height × length), membrane characteristics: material: Polyethylene terephthalate (PET), thickness: 19 µm, pore size: 5 µm, pore density of 6 × 10^4^ cm^−2^) and can be seeded with endothelial cells (EC); lower channel represents reservoir for transmigrating tumor cells (lower channel dimensions 1 mm × 1 mm × 6.1 cm (width × height × length)). Medium, tumor cells and adhesion inhibitors can be introduced to the upper channel. Homing factors and attractants can be introduced to the lower channel. B: Side view of the microfluidic device. The upper channel acts as vessel equivalent and is seeded with endothelial cells. Tumor cells can be introduced with a flow speed of 0.0 to 1.2 µL/s (correspond to 0 to 24 mm/s); all experiments were conducted with a flow velocity of 8 mm/s, as this best represents the flow velocity in small arterioles, ranging from two to 20 mm/s [48]. Vessel associated steps of metastasis (distribution (1), rolling (2), adhesion, (3) and transendothelial migration, (4) can be monitored (modified from [47])).

The adhesion of circulating tumor cells to the endothelium is essential for successful metastasis [2,3] and α_v_β_3_, α_v_β_5_ integrins together with selectins and members of the immunoglobulin family are described to play an important role in this interaction [1,2,3,5,6,9]. Therefore, the effect of Cilengitide and Cimetidine on cell adhesion of tumor cells was analyzed in an endothelial cell lined microfluidic device (Figure 1A) with and without flow conditions, mimicking vessel associated steps of metastasis (Figure 1B).

### 3.1. Cilengitide-Mediated Adhesion Inhibition

**Figure 2:**
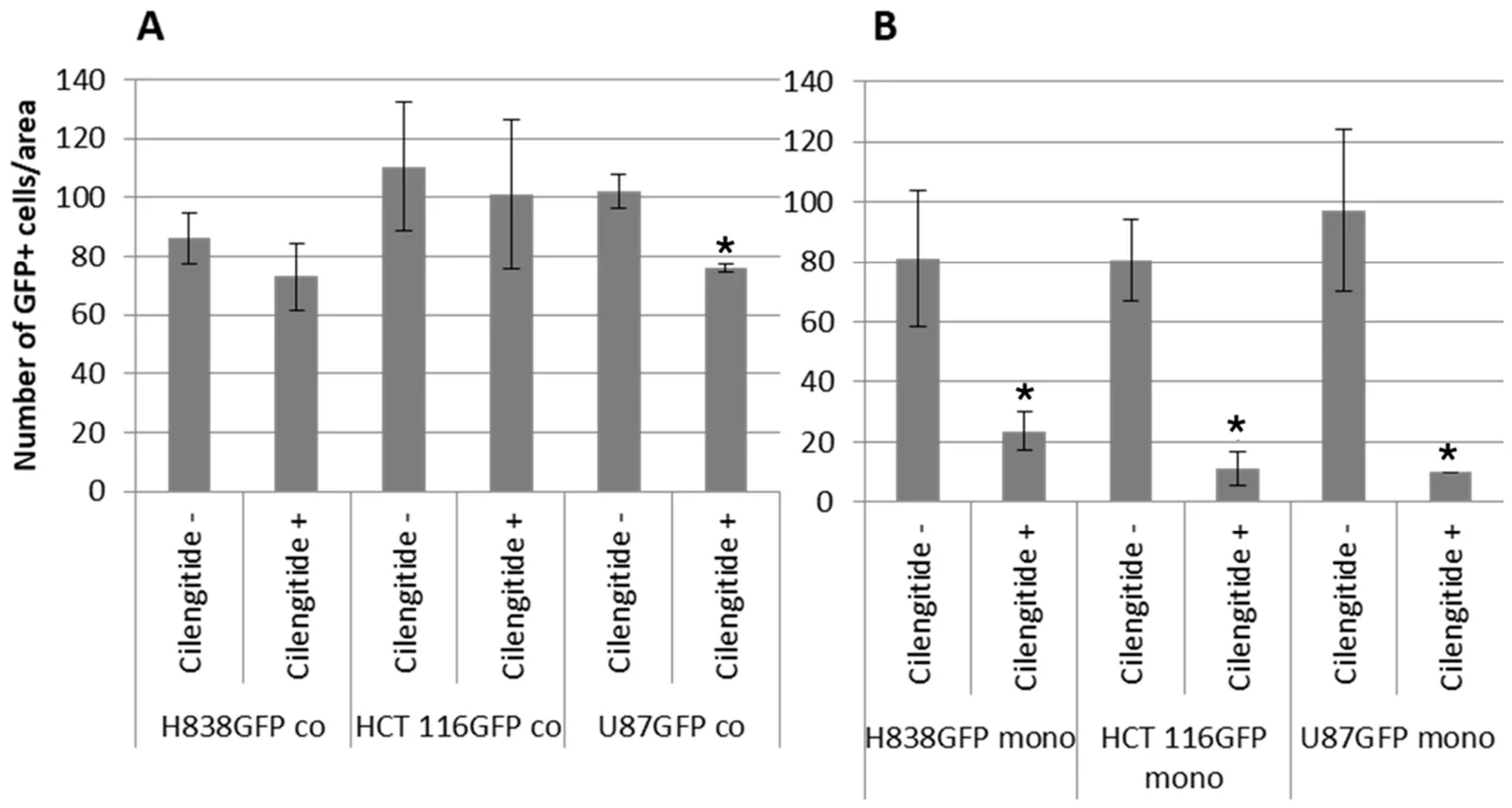
Diagrams showing the number of counted GFP positive tumor cells adherent to the porous membrane of the microfluidic device under static conditions depending on Cilengitide. (**A**): When Cilengitide was added to the single cell suspension before seeding them into the microfluidic device, populated with HPAEC endothelial cells (confluent monolayer) (Cilengitide+), the adhesion of all three tested tumor cell lines, namely H838GFP, HCT 116GFP, and U87GFP, was not inhibited (**A**). (**B**): When Cilengitide was added to the single cell suspension before seeding them into the microfluidic device with the plain porous membrane (Cilengitide+), Cilengitide significantly reduced tumor cell adhesion. Cilengitide-: no Cilengitide, control; Cilengitide+: 340 µM Cilengitide added to the cell suspension (*n* = 2). Error bars indicate SD. Statistical *t*-test compared to no Cilengitide-, *: *p* < 0.05.

The impact of Cilengitide was analyzed in the vessel-like microfluidic device in mono- as well as coculture settings using three different GFP transfected tumor cell lines. For this purpose, the upper channel and the membrane that is sandwiched in between the two parallel channels of the chip were either left without cells or seeded with HPAEC endothelial cells as described before [47]. Singularized tumor cells were injected under static conditions into the microfluidic device and incubated for 1 h. GFP-positive adherent tumor cells were then counted either on the plain membrane or on the membrane populated with endothelial cells. Cilengitide strongly inhibited tumor cell adhesion in the monoculture setup. However, when the tumor cells were injected into an endothelial cell lined device this inhibition was almost fully abolished (Figure 2A for coculture assay, Figure 2B for monoculture assay).

**Figure 3:**
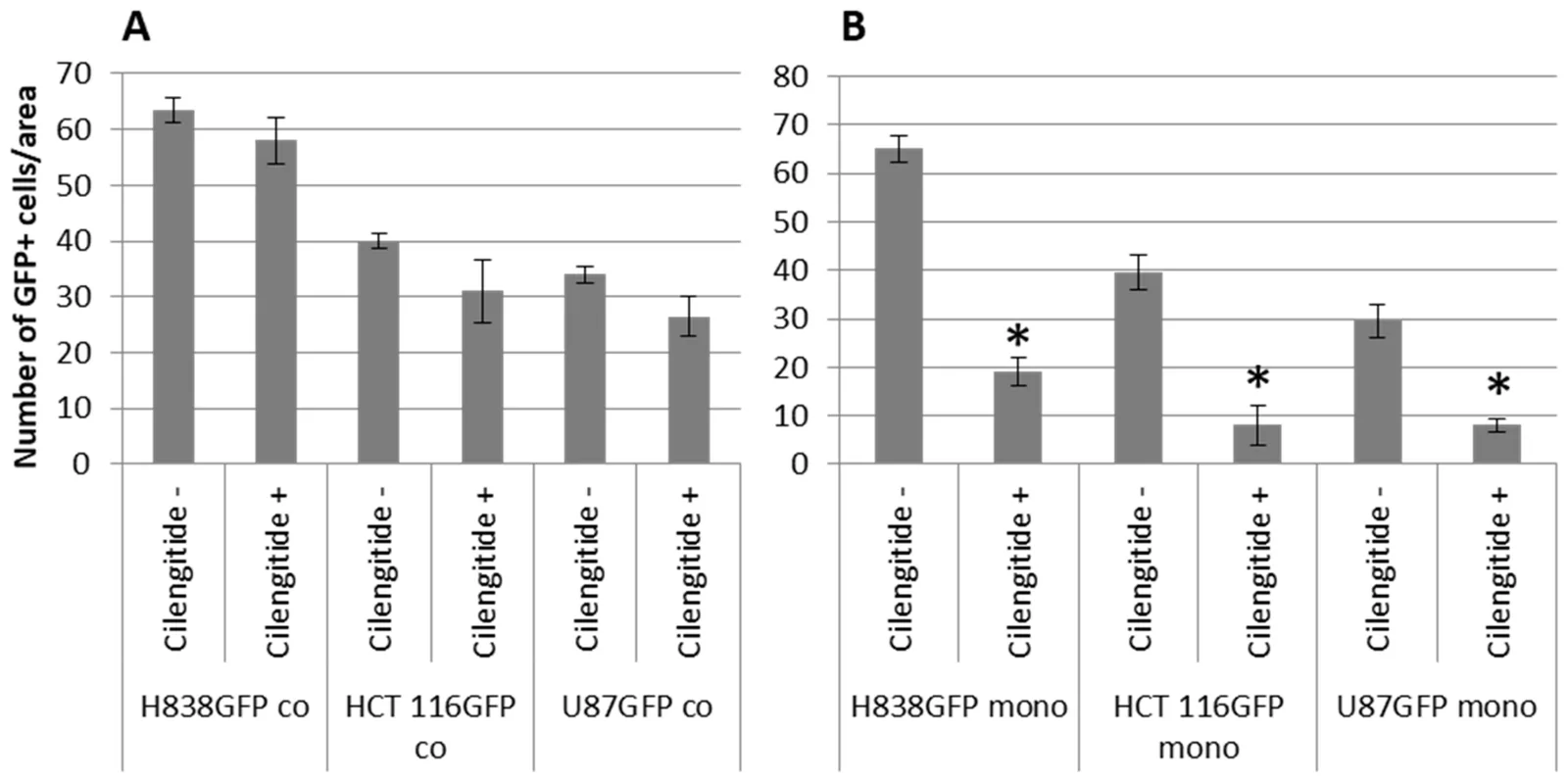
Number of counted GFP positive H838GFP lung carcinoma cells, HCT 116GFP colon carcinoma cells and U87GFP glioblastoma cells in the upper channel of microfluidic device, adherent on top of the porous membrane, either without cells (**B**) or seeded with HPAEC endothelial cells (confluent monolayer) (**A**). A total of 1 × 10^6^ tumor cells per ml were added under flow conditions with a velocity of 8 mm/s. When Cilengitide is added to the single cell suspension before seeding them into the microfluidic device (Cilengitide+), the inhibiting effect of Cilengitide is very much reduced in coculture setup with endothelial cells. When tumor cells are seeded into the microfluidic device in monoculture, Cilengitide significantly inhibits tumor cell adhesion (**B**). Cilengitide-: no Cilengitide, control; Cilengitide+: 340 µM Cilengitide added to the cell suspension (*n* = 2). Statistical *t*-test compared to Cilengitide-, Error bars indicate SD, *: *p* < 0.05.

The impact of Cilengitide on the adhesion of H838GFP lung carcinoma cells was additionally evaluated under flow conditions with a flow velocity of 8 mm/s (Figure 3A coculture setup, Figure 3B monoculture setup).

As already demonstrated under static conditions, also under dynamic flow conditions in the microfluidic device, Cilengitide-mediated inhibition of tumor cell adhesion could only be observed in the absence of endothelial cells in the microfluidic device. When tumor cells were introduced into an endothelial cell lined device Cilengitide had no significant influence on tumor cell adhesion.

**Figure 4:**
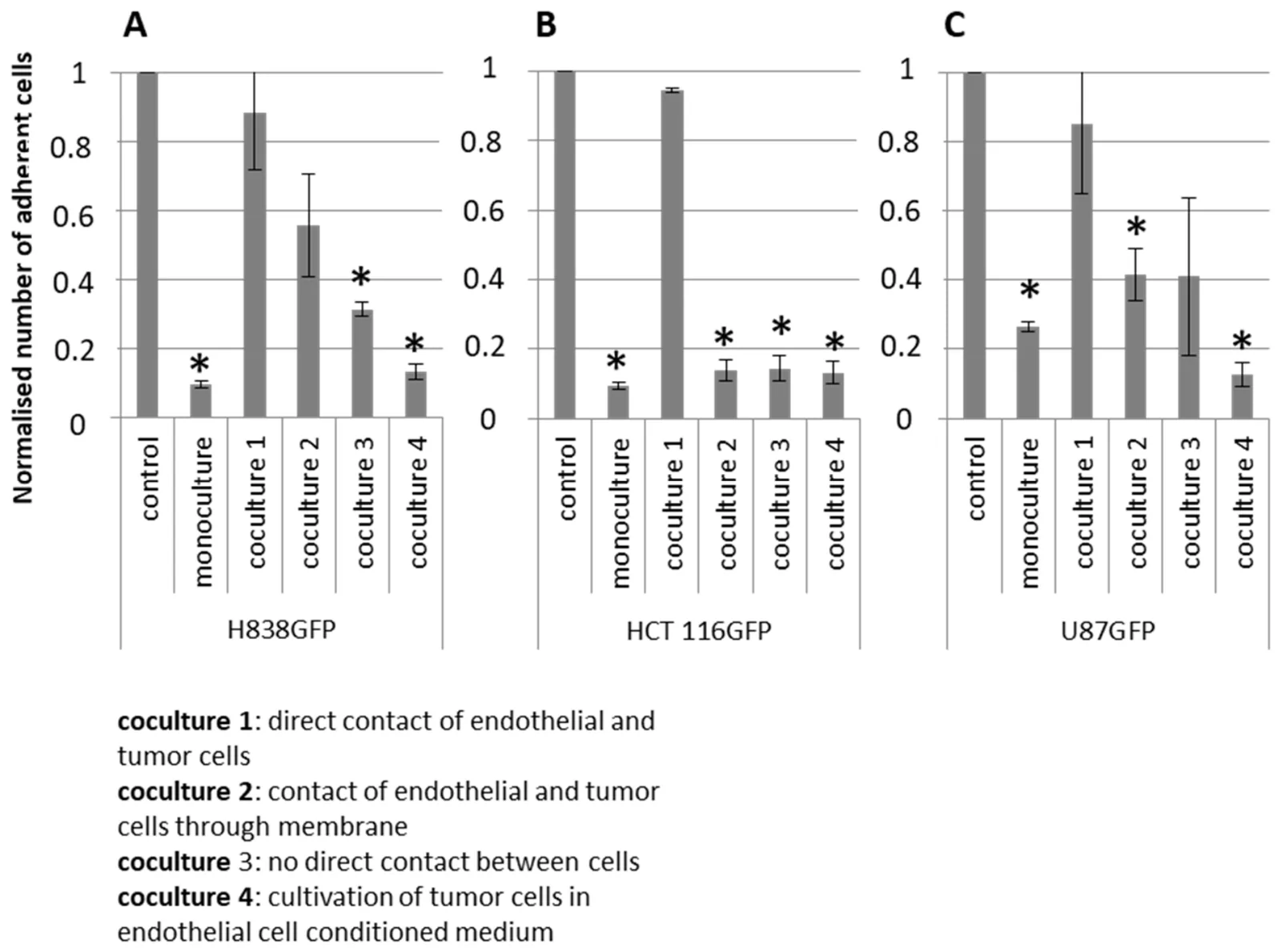
Number of adherent tumor cells (normalized to control) in tumor cell monocultures or different coculture settings with HPAEC endothelial cells after treatment with Cilengitide 340 µM. Coculture 1: endothelial and tumor cells seeded in the upper Boyden Chamber compartment; coculture 2: endothelial cells on the bottom of insert membrane, tumor cells in upper Boyden Chamber compartment; coculture 3: endothelial cell in lower compartment, tumor cells in upper Boyden Chamber compartment; coculture 4: tumor cells in upper compartment in the presence of endothelial cell conditioned medium (*n* = 3). Cell numbers are normalized to control. In monocultures, Cilengitide shows a strong inhibitory effect on the adhesion of H838GFP (**A**), HCT116GFP (**B**) and U87GFP cells (**C**). In coculture assays with H838GFP cells (**A**), the inhibiting effect of Cilengitide is reduced depending on the amount of contact between tumor cells and coculture’s endothelial cells. When tumor cells are cultivated in endothelial cell conditioned medium, the inhibiting effect of Cilengitide is reduced, compared to the monoculture assay. (**B**) In the assays with HCT116GFP cells (**B**), coculture setting 1 involves direct physical contact between tumor and endothelial cells, and the adhesion inhibiting effect of Cilengitide is almost completely abolished. In the other coculture setups without cell–cell contacts, Cilengitide dependent adhesion inhibition is maintained. (**C**) In the coculture setting with U87GFP cells (**C**), the inhibiting effect of Cilengitide on the adhesion is strongly reduced, when endothelial and tumor cells are in direct contact with each other in the upper Boyden Chamber compartment. In cocultures 2 and 3 with almost no or no direct contact between the two cell types, Cilengitide inhibits cell adhesion albeit to a significantly lower extent than in the monoculture assay. In coculture 4 with conditioned media only, Cilengitide has similar inhibiting effects than in the monoculture assay. Error bars indicate SD. Statistical *t*-test compared to control, *: *p* < 0.05.

To determine whether the lack of Cilengitide efficacy was the result of a direct contact of tumor cells to the endothelial cell monolayer or was simply dependent on the presence of endothelial cells in the system, a Boyden Chamber Assay was performed. Three different GFP transfected tumor entities (H838GFP lung carcinoma (Figure 4A), HCT116GFP colon carcinoma (Figure 4B), and U87GFP glioblastoma cells (Figure 4C)) were employed, either in monoculture or in coculture settings with HPAEC endothelial cells. To ensure a confluent endothelial monolayer for the coculture experiments, HPAECs were seeded one day before the tumor cells in three different positions within the Boyden Chamber, either in the upper compartment on top of the membrane (coculture 1), or below the membrane (coculture 2) or in the bottom compartment (coculture 3). Additionally, conditioned media of separately cultured endothelial cells was employed (coculture 4). Tumor cells either untreated or treated with 340 µM of Cilengitide were added to the upper compartment of the Boyden Chamber. Figure 4 shows the evaluation of the number of adherent tumor cells in monoculture and in the different coculture settings. The results of the monoculture assay in the Boyden Chamber confirm those obtained in the microfluidic device. Cilengitide had a strong inhibiting effect on cell adhesion of H838GFP, HCT 116GFP, and U87GFP in monoculture. In the coculture assays, an inhibiting effect of Cilengitide on tumor cell adhesion could only be observed when there was no direct contact between endothelial cells and tumor cells. The most pronounced inhibitory effect of Cilengitide was seen on H838GFP and U87GFP cells in cultures where only HPAEC conditioned media was employed. In cultures with H838GFP and similarly also with U87GFP tumor cells Cilengitide-dependent inhibition of tumor cell adhesion decreased with increasing physical contact between tumor cells and endothelial cells. In coculture three, where HPAEC endothelial cells were grown on the bottom of the lower compartment tumor cells did not have direct contact to the vascular cells but could communicate with endothelial cells via cytokines or other soluble mediators. For H838GFP tumor cells, the inhibitory effect of Cilengitide was reduced in this setting in comparison to the experiment where only endothelial cell conditioned media was used. Even less inhibition was seen in coculture setting two, where endothelial cells and tumor cells were in contact with each other through the pores of the Boyden Chamber membrane. Finally, no inhibiting effect could be observed in coculture one, were direct cell–cell contact between endothelial and tumor cells was permitted. These data suggest that intimate physical contact between tumor cells and endothelial cells abolishes Cilengitide mediated adhesion inhibition.

In the adhesion assays with HCT 116GFP cells (Figure 4B), again maximal Cilengitide-dependent adhesion inhibition was achieved in monoculture. In cocultures only direct contact of tumor cells with the endothelial cells (coculture setting 1) clearly abolished the Cilengitide effect. All other coculture settings with limited or no direct contact between tumor and endothelial cells show a Cilengitide dependent inhibition of tumor cell adhesion.

**Figure 5:**
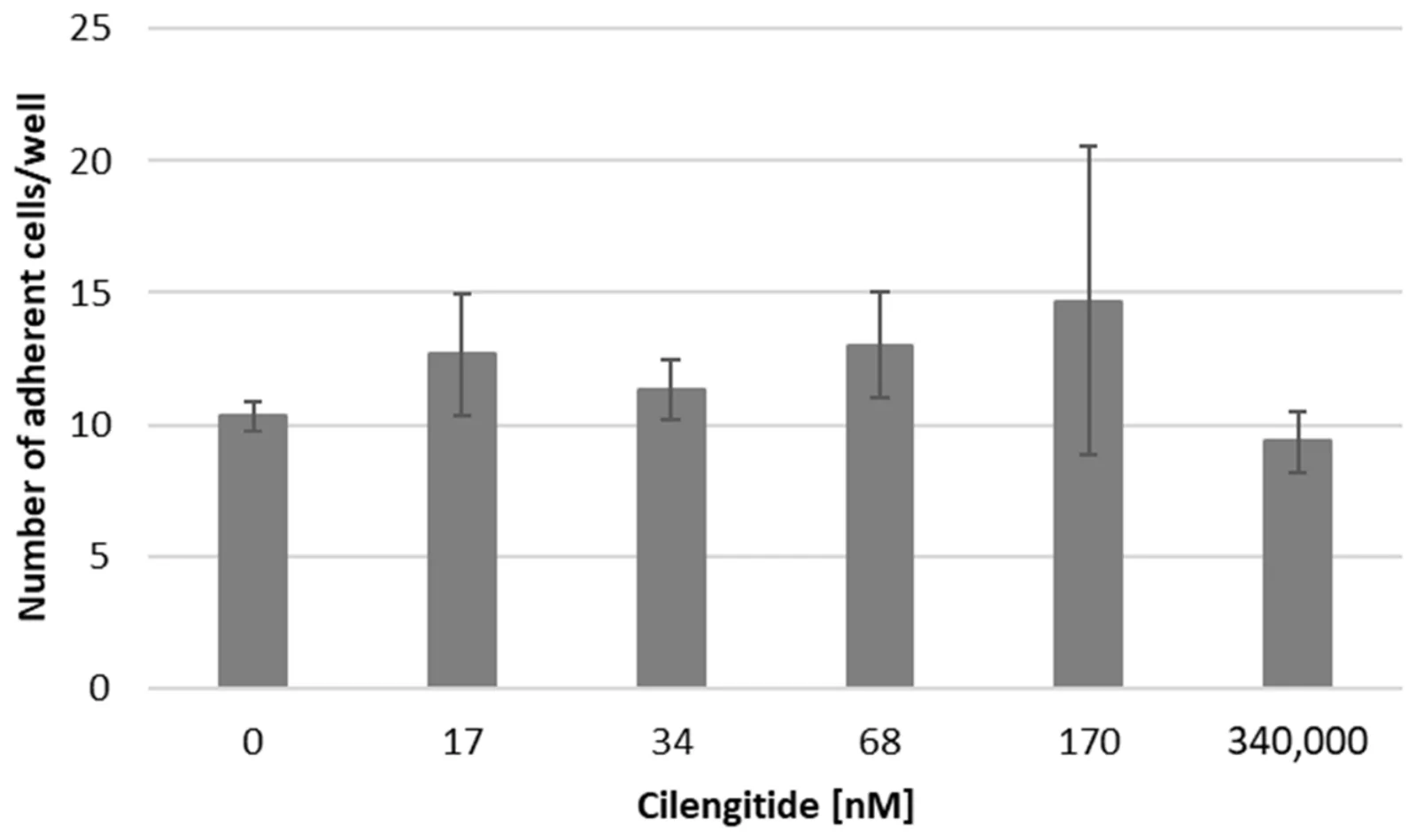
Diagram comparing the number of H838GFP cells adhering to a monolayer of HPAEC endothelial cells after treatment with different concentrations of Cilengitide. Independent of the Cilengitide concentration there is no significant influence of the integrin inhibitor on cell adhesion (*n* = 3). Statistical *t*-test compared to control (0 nM), Error bars indicate SD.

To determine whether this difference in inhibition efficacy between the mono- and coculture setting could be abolished by increasing the Cilengitide concentration in the experiments simple cell adhesion assays, were conducted in tissue culture plates with tumor cells in monoculture or in cocultures of tumor cells with HPAEC endothelial cells. The impact of different concentrations of Cilengitide on the adhesion H838GFP lung carcinoma cells on a HPAEC endothelial cell monolayer is summarized in Figure 5.

Independent of the concentration used, Cilengitide had no significant influence on the number of tumor cells adhering to the endothelial cell layer.

**Figure 6:**
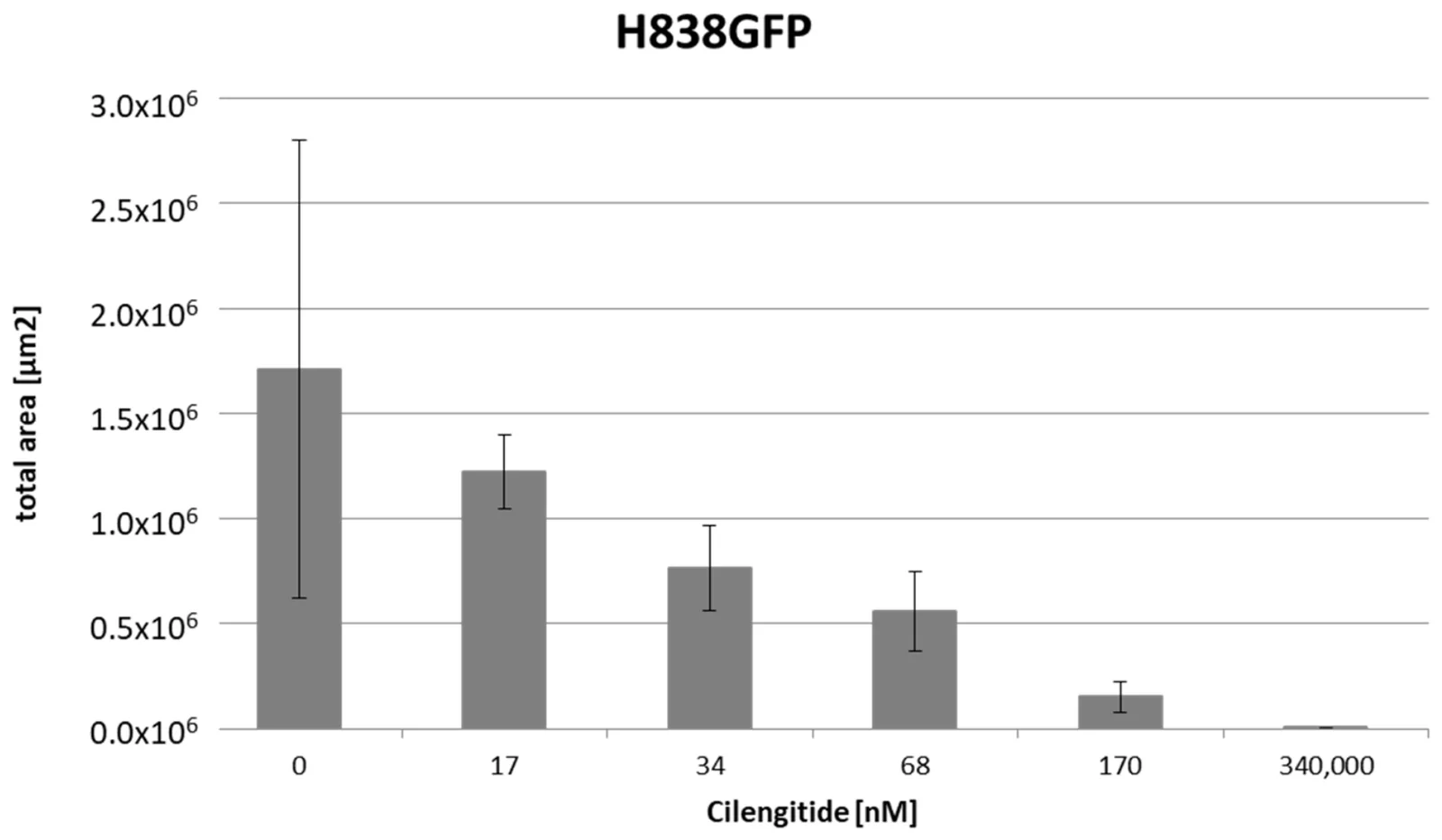
Diagram comparing H838GFP cells adhesion, in monocultures treated with different concentrations of Cilengitide. The columns represent the total area of adherent cells. With increasing concentrations of Cilengitide, the number of adherent cells decreases in a monocultural setup (*n* = 3). Statistical *t*-test compared to control (0 nM), Error bars indicate SD.

In contrast, in the cell adhesion assay in monocultures of H838GFP lung carcinoma cells Cilengitide significantly inhibited tumor cell adhesion in a concentration dependent manner. At a concentration of 340 µM almost no tumor cells adhered to the culture plate (Figure 6).

To determine whether this lack of Cilengitide-mediated inhibition of tumor cell adhesion could be exclusively attributed to integrins α_v_β_3_ and α_v_β_5_, the inhibition of another integrin described to be involved in adhesion processes to vascular endothelium, the α_4_β_1_ integrin, was analyzed (4). The impact of an α_4_ inhibitory antibody was determined on the adhesion of H838GFP cells in monoculture and coculture with HPAEC endothelial cells. No inhibition of tumor cell adhesion neither in monocultures nor in cocultures on top of HPAEC endothelial cells could be achieved by inhibiting the α_4_ integrin (Supplementary Materials File). Thus, while the inhibitory effect on tumor cell adhesion in monoculture is clearly specific for the α_v_β_3_ and α_v_β_5_ integrin inhibitor Cilengitide. The inhibition is significantly reduced and even abolished in the presence of endothelial cells. This holds especially true for cocultures where tumor cells are in direct cell–cell contact with endothelial cells, i.e., are required to directly adhere to an endothelial cell monolayer similar to the situation in the blood vessel in vivo. In this setting the inhibitory effect of Cilengitide is abolished in all three tumor types tested, indicating that the adhesive behavior of tumor cells in the presence of the integrin inhibitor Cilengitide (340 µM) strongly depends on architectural conditions and cell–cell contacts. These results suggest that integrins are not the primary mediators of this first vessel associated step of metastasis.

Consequently, the role of selectin, an important mediator of leukocyte rolling and extravasation was analyzed in the adhesion and transendothelial migration of tumor cells.

### 3.2. Cimetidine-Mediated Extravasation Inhibition

Cimetidine, a registered treatment for gastric ulcer diseases and gastro esophageal reflux [40,41] has been described to influence cell adhesion, invasion, angiogenesis and proliferation in different tumor entities due to its effect as E-selectin inhibitor [42,43,44,45]. Therefore, the influence of Cimetidine on tumor cell adhesion was analyzed.

**Figure 7:**
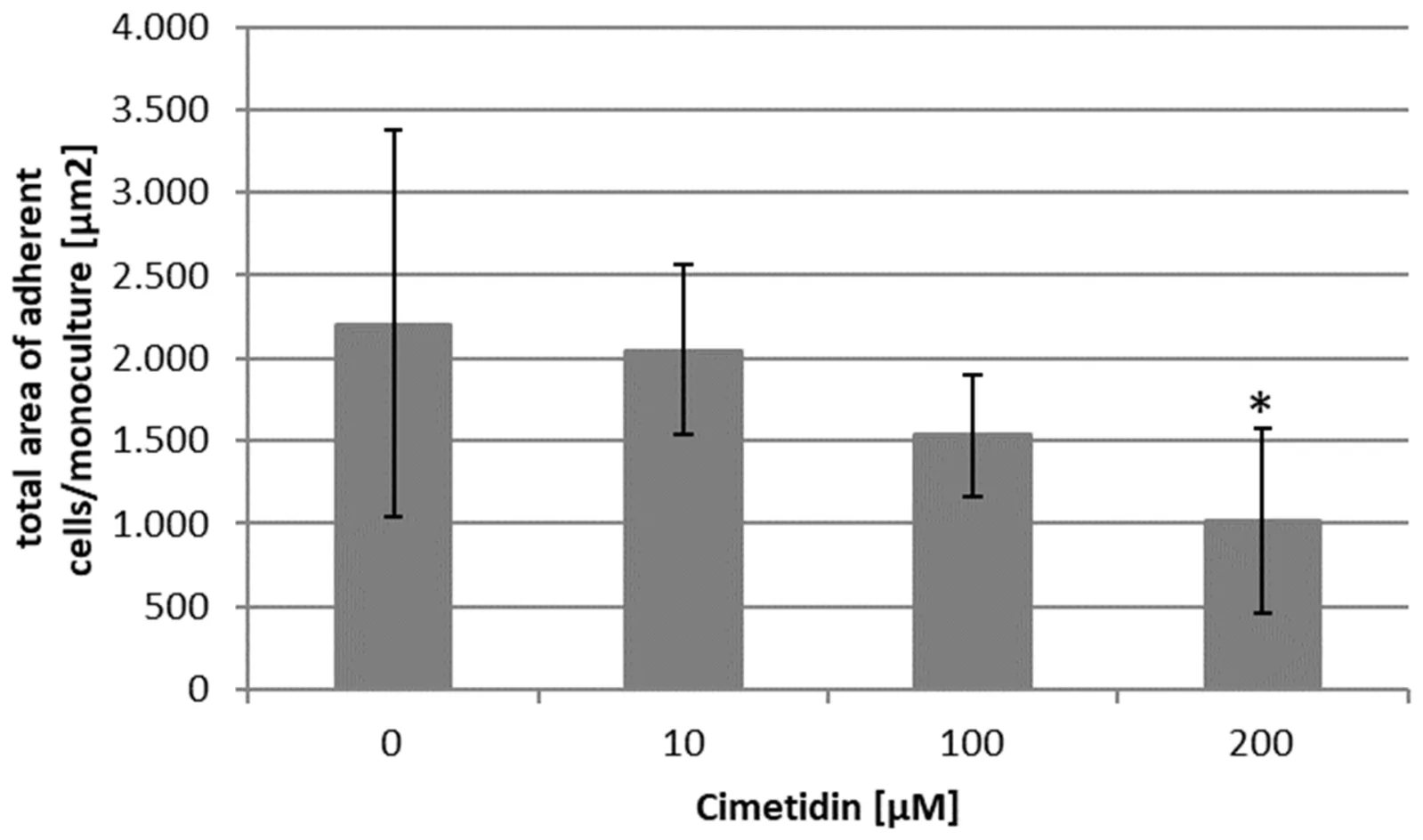
Diagram representing the total area of adherent H838GFP cells in monoculture, when treated with different concentrations of Cimetidine. Results show a decrease in H838GFP cells adhesion with increasing concentration of Cimetidine. (*n* = 6). Error bars indicate SD. Statistical *t*-test compared to control (0 µM), *: *p* < 0.05.

**Figure 8:**
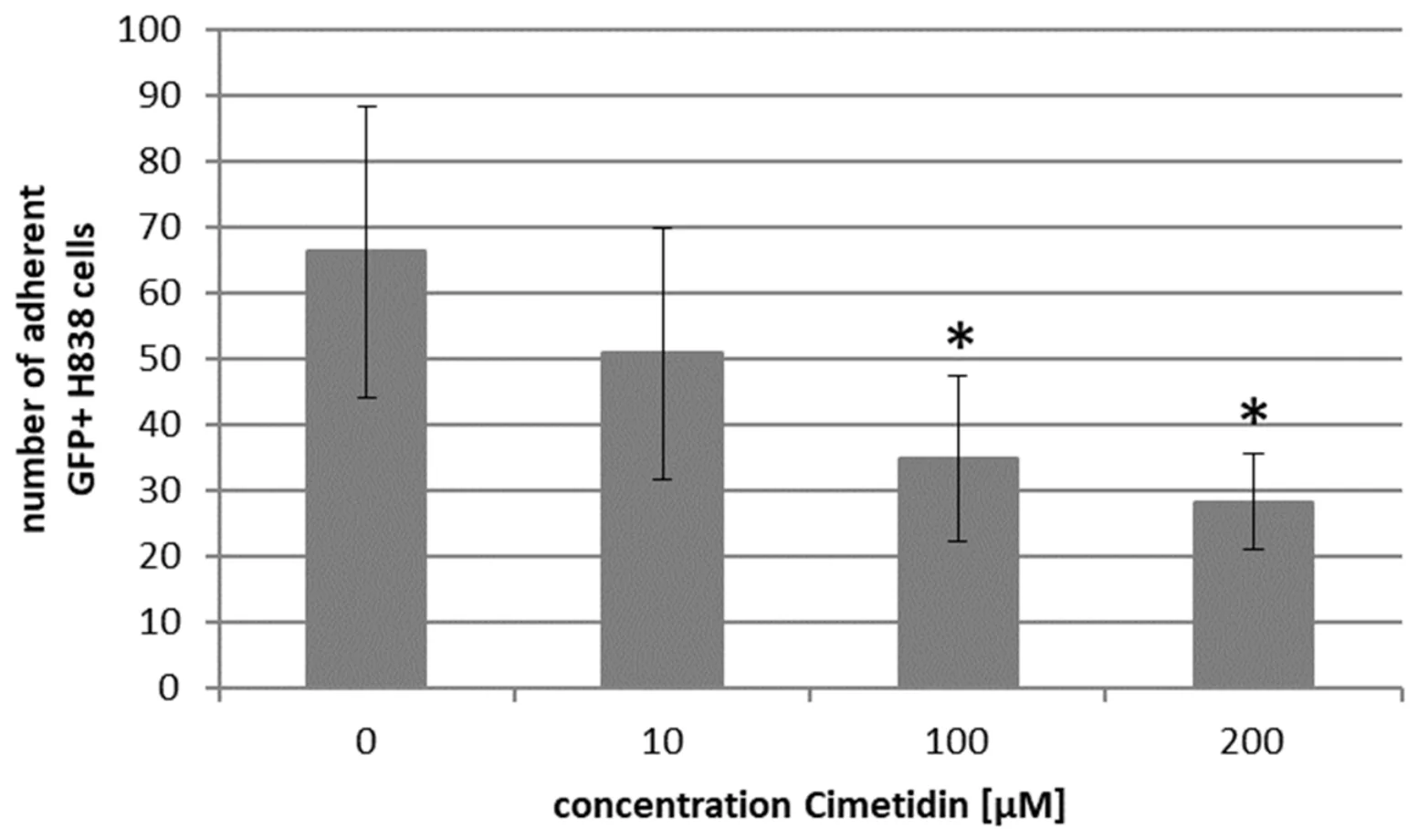
Diagram representing the total number of adherent H838GFP cells in coculture on a HPAEC monolayer when treated with different concentrations of Cimetidine. Results show a concentration dependent decrease in adherent H838GFP cells. (*n* = 6). Error bars indicate SD. Statistical *t*-test compared to control (0 µM), *: *p* < 0.05.

In the first step Cimetidine treatment was tested in different concentration in adhesion assays with either H838GFP alone (Figure 7) or H838GFP seeded on a HPAEC endothelial cell monolayer (Figure 8).

Cimetidine-induced a significant reduction in cell adhesion for both H838GFP tumor cells in monoculture, as well as in coculture on a monolayer of HPAEC endothelial cells. Inhibition occurred in a concentration dependent manner. For further experiments Cimetidine was used in a concentration of 200 µM as it shows a reduction in adherent tumor cells by more than 50% compared to controls.

**Figure 9:**
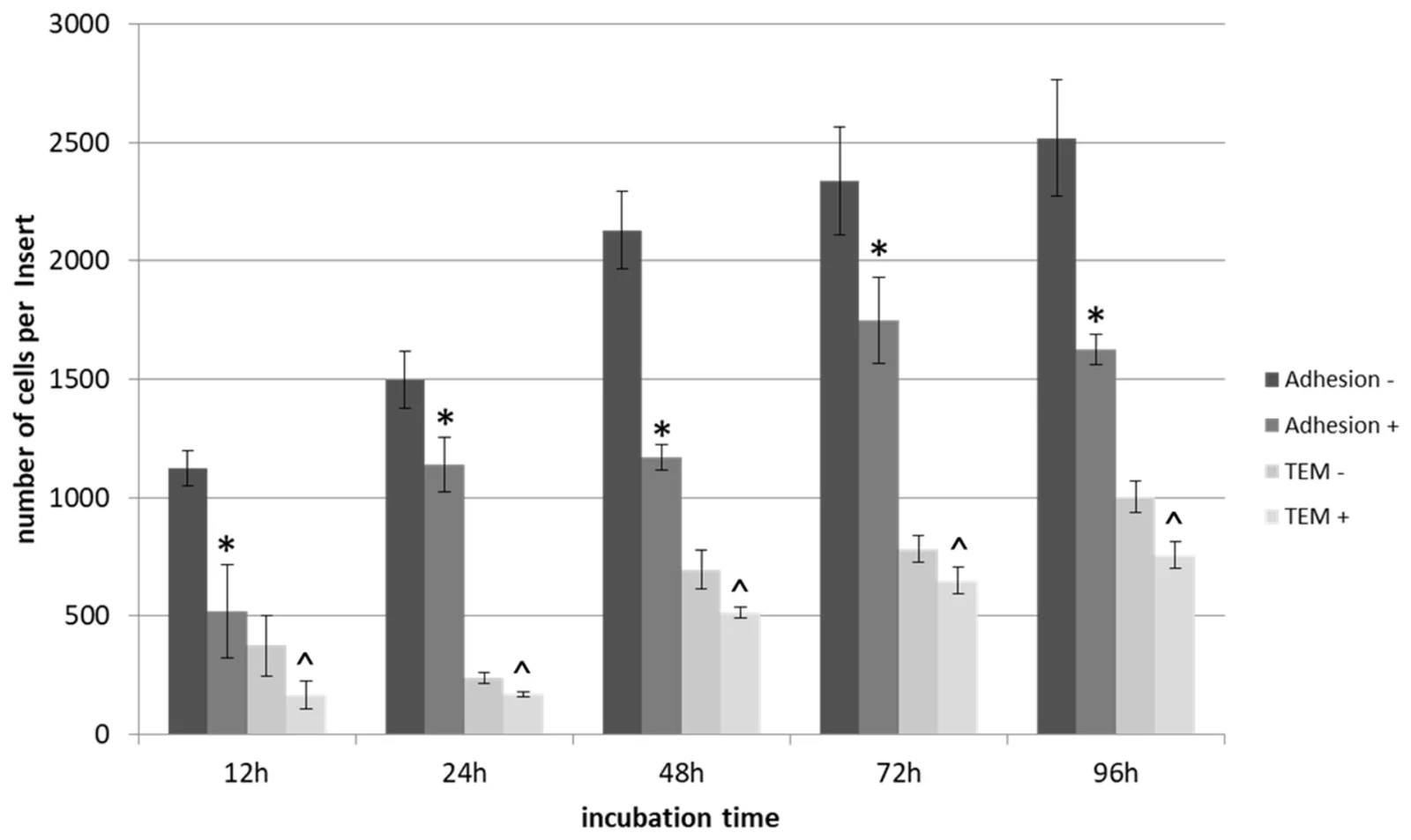
Effect of 200 µM Cimetidine on the number of adherent and transmigrating (TEM) H838GFP cells through a HPAEC monolayer in a Boyden Chamber. Results show a decrease in both H838GFP adherence and transmigration upon Cimetidine treatment. -: 0 µM Cimetidine, +: 200 µM Cimetidine (*n* = 3). Adhesion-: number of adherent cells without treatment. Adhesion+: number of cells adherent with treatment. TEM-: number of cells transmigrated through the HPAEC monolayer without treatment. TEM+: number of transmigrated cells with treatment. Error bars indicate SD. Statistical *t*-test compared to without treatment (Adhesion- or TEM-), *: *p* < 0.05 (Adhesion), ^: *p* < 0.05 (TEM).

To further characterize the impact of 200 µM Cimetidine on tumor cell adhesion H838GFP cells were treated and seeded on top of an endothelial monolayer in a Boyden Chamber assay. Tumor cell adhesion and transendothelial migration were determined (Figure 9) after an experimental time of 96 h. Cimetidine treatment resulted in a significant decrease in the number of adherent as well as transmigrating tumor cells by at least 25% and 10%, respectively.

**Figure 10:**
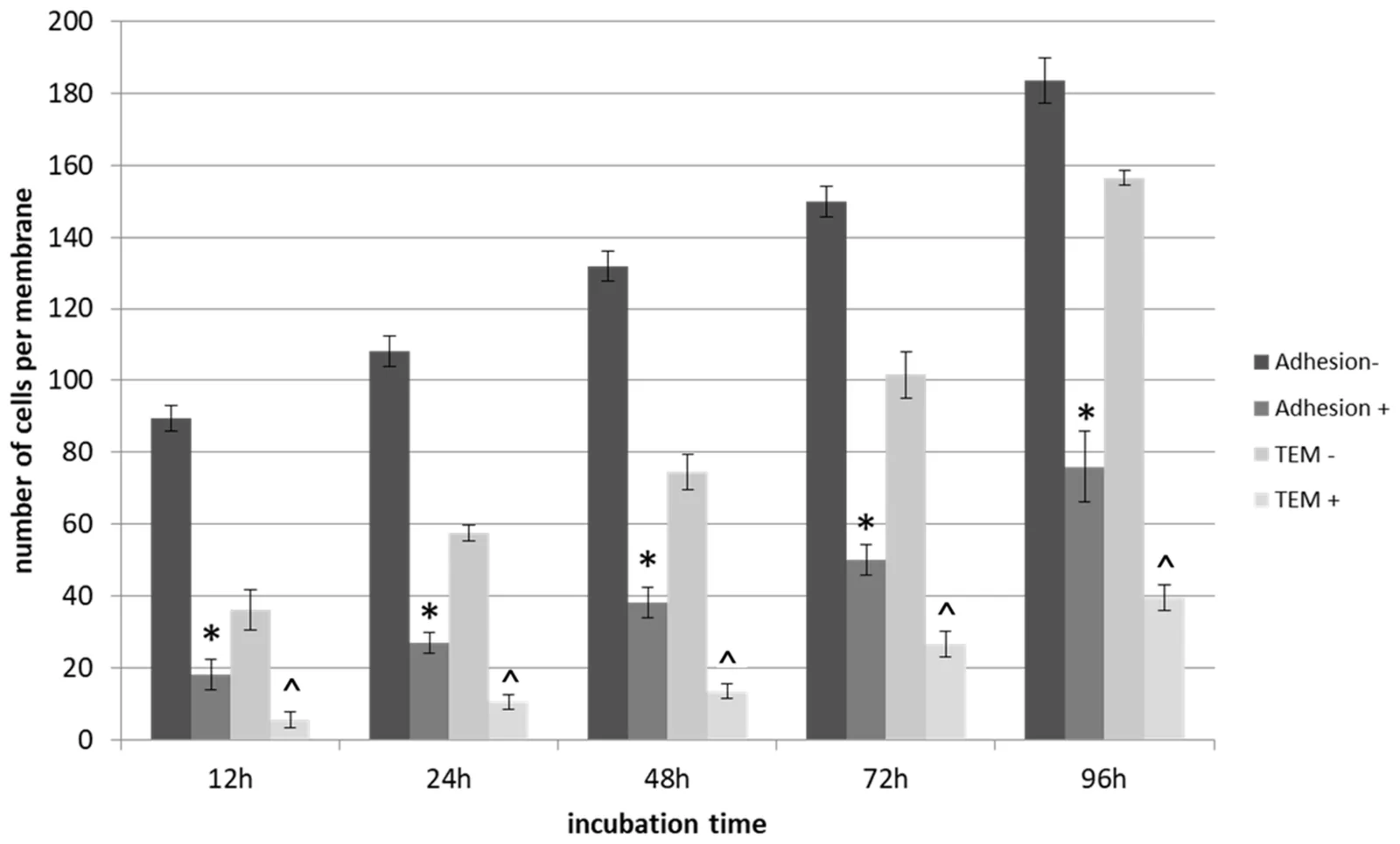
Number of adherent and transmigrated (TEM) H838GFP cells through a HPAEC monolayer in the static microfluidic device when treated with 200 µM Cimetidine. Results show a decrease in both numbers of adherent and transmigrated H838GFP cells. w/o: 0 µM Cimetidine, w/: 200 µM Cimetidine (*n* = 2). Error bars indicate SD. Statistical *t*-test compared to without treatment (Adhesion- or TEM-), *: *p* < 0.05 (Adhesion), ^: *p* < 0.05 (TEM).

To confirm these results in a more physiological vessel-like setting, the effect of 200 µM Cimetidine on adhesion and transendothelial migration of H838GFP cells was also tested in the static microfluidic device (Figure 10). During an experimental time of 96 h, the number of cells adherent to the confluent HPAEC endothelial cell monolayer lining the microfluidic channel significantly decreased by at least 65% upon Cimetidine treatment. Similarly, the number of transmigrating H838GFP cells was significantly reduced by at least 70% at all time points tested.

**Figure 11:**
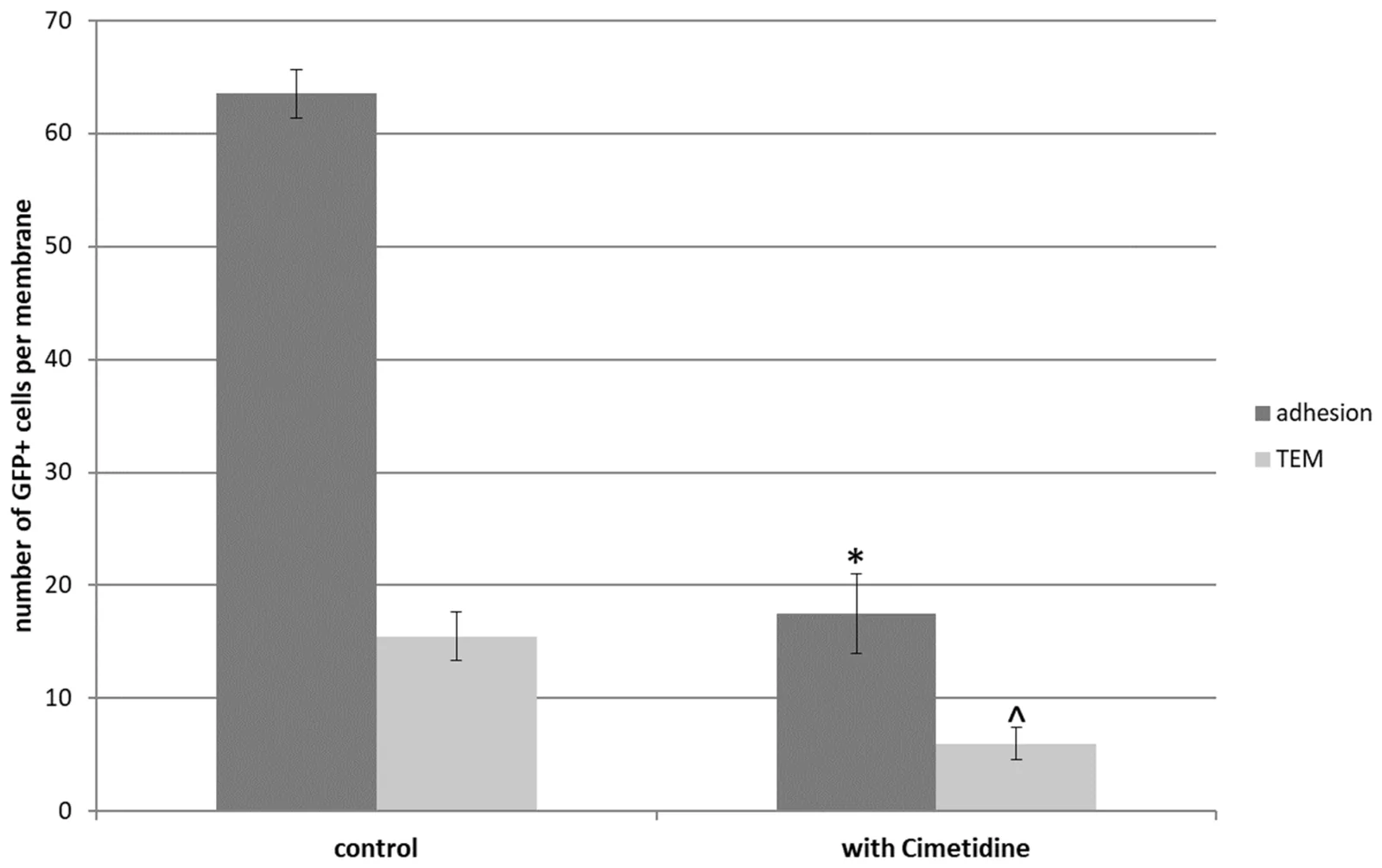
Impact of 200 µM Cimetidine on the number of H838GFP cells adherent on a confluent HPAEC monolayer and transmigrating (TEM) through the endothelial lining in the dynamic microfluidic device. Cell numbers of both adherent and transmigrated H838GFP cells are decreased. Experimental time: 24 h. Flow velocity: 8 mm/s (*n* = 2). Error bars indicate SD. Statistical *t*-test compared to control (without treatment, *: *p* < 0.05 (Adhesion), ^: *p* < 0.05 (TEM).

Finally, to determine whether Cimetidine is also capable of inhibiting tumor cell adhesion to endothelial cells and transendothelial migration under dynamic flow conditions and thus could exert its effect also in an in vivo environment, tumor cells treated with 200 µM Cimetidine were introduced into the microfluidic device at a flow velocity of 8 mm/s. During 24 h the treatment of H838GFP cells with Cimetidine resulted in a significant decrease by 70% of cells adherent to and transmigrating through the HPAEC endothelial cell lining of the vessel channel (Figure 11).

Taken together, the data underline the importance of the physiological tissue context when analyzing therapeutic efficacy. They demonstrate a lack of efficacy for the integrin inhibitor Cilengitide in the inhibition of tumor cell adhesion that is mediated by the presence of and cell–cell contacts to microvascular endothelial cells. In contrast inhibition of E-selectin by Cimetidine significantly reduced adhesion and transendothelial migration of tumor cells thereby inhibiting this crucial step in the metastatic process. These data suggest a potential benefit of Cimetidine in the treatment of metastatic dissemination of tumor cells.

## 4. Discussion

One critical step for successful establishment of metastases in distant organs is the adhesion and subsequent transendothelial migration of circulating tumor cells to the endothelial lining of blood vessels [1,2]. This interaction is thought to be mediated by integrins and proteases [3,4]. Integrins function as adhesion molecules, signal transducers and mechanosensors [10,17]. The expression of the integrins α_v_β_3_, α_v_β_5_, α_5_β_1_, α_6_β_4_ as well as of the α_4_ subunit can be correlated with tumor progression in different cancers [12,17,18,19]. Integrins α_v_β_3_ and α_v_β_5_ are responsible for cell adhesion, migration, proliferation of tumor cells and thought to be involved in tumor metastasis. Specifically, they are described to be responsible for the arrest of tumor cells on the endothelium cells of blood vessel, and as such for tumor cell extravasation and metastasis [3,11,19,26].

Activation of these integrins can be selectively blocked by Cilengitide [7,8,22] suggesting a potential use of this substance in cancer therapy. Indeed, treatment with Cilengitide seemed to inhibit tumor cell adhesion in monocultures of tumor cells and simple coculture set ups [4,10,11,23,24,25,26,27]. Yet clinical studies show only limited or no beneficial effects of Cilengitide on patient survival [14].

To elucidate the reason for this discrepancy and to determine potential therapeutic alternatives, we took advantage of our microfluidic metastases model mimicking capillary physiology in the context of vessel associated steps of metastases. Cilengitide was used in this study as selective blocker of the integrins α_v_β_3_ and α_v_β_5_ [7,8], and its effect on tumor physiology and tumor cell adhesion to and transmigration through an endothelial cell lined vessel structure was determined. Cilengitide does not inhibit tumor cell migration of the tumor cell line H838GFP used in this study (Supplementary Materials File). However, its effect on the adhesion of different tumor cell lines was analyzed either in monoculture on tissue culture surfaces or on a confluent monolayer of endothelial cells. In the monoculture setup, treatment with Cilengitide resulted in a strong inhibition of tumor cell adhesion up to 90% as was described by others [7,18,19,20]. Willisch-Neumann et al. described a decreased motility and invasion at a Cilengitide concentration of 1 mg/ml [19]. In this study, a considerably lower concentration of 340 µM Cilengitide already resulted in a strong inhibition of tumor cell adhesion in monoculture experiments. In agreement with previous studies on breast cancer cells [11], osteosarcoma cells [25], malignant pleural mesothelioma cells [27], as well as prostate cancer, melanomas and gliomas [10] increasing concentration of Cilengitide, lead to a decreasing number of adherent cells in monoculture.

However, the Cilengitide-dependent inhibition of tumor cell adhesion could not be recapitulated in an in vivo like setting tumor cells came in physical contact with HPAEC endothelial cells lined vessel wall in a microfluidic model for vessel associated steps of metastasis [47]. In this setting Cilengitide had no effect on tumor cell adhesion. Interestingly the lack of adhesion inhibition was not exclusively mediated by direct cell–cell contact between tumor cells and endothelial cells but could also be observed, albeit to a reduced extend, when tumor cells and endothelial cells had only minimal contact through the membrane of a Boyden chamber or were interacting solely by soluble factors. Similar results were previously shown in rats, where the blockage of β_1_-, β_4_-, and α_v_- integrins could not inhibit colon carcinoma cell adhesion in the lung [9]. On the other hand, there are studies describing an inhibition of adhesion of non-small lung cancer cells on umbilical vein endothelial cells after treatment with Cilengitide in combination with radiotherapy [21]. Additionally, in a xenograft mouse model, treatment with Cilengitide showed suppression of metastatic growth but exhibited only a minimal effect on the growth of the primary tumor [25]. In a cell culture model with brain tumor cells and brain capillary endothelial cells, treatment with Cilengitide lead to detachment and apoptosis of both endothelial and brain tumor cells [21]. Such effects of Cilengitide on the HPAEC endothelial cell monolayers could not be confirmed in this study (Supplementary Materials File). In addition, treatment of a 3D tumor stroma model containing tumor cell spheroids in a dextran hydrogel with microvascular endothelial cells, fibroblasts, macrophages, and neutrophil [49] did not show any effect of Cilengitide either on tumor spheroid growth nor on the cells in the stromal compartment or the angiogenic sprouting of vascular structures (Supplementary Materials File).

The results shown in this study, confirm that the integrins α_v_β_3_, α_v_β_5_ play a role in the adhesion of tumor cells in a monoculture setup of H838 lung carcinoma cells, HCT 116 colon carcinoma cells and U87 glioblastoma cells. This adhesion can be blocked in a concentration-dependent manner by the integrin inhibitor Cilengitide. However, the adhesion of tumor cells to a HPAEC endothelial monolayer cannot be inhibited by Cilengitide for any of the tumor entities tested, and there are even indications that an interaction of tumor cells with endothelial cells solely via soluble factors or with only minimal physical contact might interfere with Cilengitide efficacy (data shown for H838). Taken together these data demonstrate the importance of a physiological tissue environment when testing therapeutic substances and suggests that the adhesion of tumor cells to the endothelial lining during the metastatic extravasation process might not be mediated by α_v_β_3_ or α_v_β_5_ integrin but rather depend on different adhesive mechanisms.

Cilengitide as a potential inhibitor of α_v_β_3_ and α_v_β_5_ integrins was used in clinical studies. However, only a phase I trial with patients suffering from melanoma and glioblastoma showed a benefit for the treatment with Cilengitide [50]. These results could not be confirmed in a phase III clinical trial. Here, patients showed no benefit and therefore no better outcome after treatment with Cilengitide [14,34] thereby putting the central role of these integrins in tumor progression and metastasis in question. The data presented here confirm such lack of efficacy for Cilengitide with respect 3D in vitro tumor-stroma cultures representing the primary tumor and its environment as well as in the context of vessel associated steps of metastasis. Besides the lack of efficacy for Cilengitide in inhibiting tumor growth and angiogenesis in an in vivo-like tumor-stroma model, they also point to integrin-independent mechanisms governing vessel associated steps of metastasis.

Selectins, another class of adhesion molecules are involved in leukocyte rolling [39] and are believed to play a role in tumor cell adhesion, migration and extravasation [1,2]. The H2-receptor antagonist, Cimetidine, also acting as E-selectin inhibitor, has been described to have effects on tumor cell proliferation, adhesion, invasion, angiogenesis in different tumor entities [40,41,43,44]. Its role as H2-receptor inhibitor might also account for part of the anti-tumor effect of Cimetidine since in some tumor malignancies, like breast, gastric cancer and melanoma cells, Histamine acts as receptor-independent growth factor, promoting proliferation [42,44,45].

In this study HCT 116GFP, U87GFP, and H838GFP lung carcinoma cells were treated with Cimetidine, employing different mono- and coculture assays. Treatment of H838GFP cells resulted in reduction in tumor cell adhesion to and transmigration through an endothelial monolayer of HPAEC. This inhibitory effect was even increased in an endothelial cell lined microfluidic capillary under flow conditions suggesting an enhanced effect of this therapeutic intervention in a physiological capillary environment. In addition, treatment of 3D tumor stroma cultures containing U87GFP and HCT 116 GFP tumor spheroids in a dextran hydrogel with HPAEC endothelial cells human fibroblasts and inflammatory cells [49] revealed an inhibitory effect of Cimetidine on the development of a tumor supporting stroma and on angiogenesis, the sprouting of vascular structures and thus ultimately also on tumor spheroid growth (Supplementary Materials File). Adhesion inhibiting effects of Cimetidine were also shown for colon and breast cancer cells on HUVEC endothelial cells in vitro [42,43,44]. Additionally, Cimetidine treatment induced an inhibition of angiogenesis by reducing vascular endothelial growth factor in mouse models [40,44]. Finally, several clinical trials confirmed a survival benefit for cancer patients after Cimetidine treatment [40,42,45].

Taken together, these data, obtained in two independent, in vivo-like physiological tissue systems (3D tumor-stroma culture and microfluidic vessel equivalent), suggest a significant role of E-selectin in tumor progression and metastasis. E-Selectin contributes to the establishment of a tumor supporting stroma with vessels sprouting towards the tumor and mediates tumor cell adhesion and transendothelial migration of H838GFP lung carcinoma cells. Consequently, this suggests Cimetidine as potentially beneficial anti-cancer drug that reduces both angiogenesis and adhesion and transendothelial migration of tumor cells. At the same time the data presented here provide a possible explanation for the lack of efficacy seen for the integrin inhibitor Cilengitide in clinical studies.

## 5. Conclusions

The results of this study give evidence to the importance of a physiological tissue context when testing therapeutic interventions in tumor progression and metastasis. They confirm that the inhibition of the α_v_β_3_, α_v_β_5_ integrins by Cilengitide massively reduces the cell adhesion of different cancer cell types to tissue culture substratum. However, they also clearly demonstrate that integrins α_v_β_3_, α_v_β_5_ are not responsible for tumor cell adhesion to endothelial cells during extravasation from the vasculature during early steps of metastasis or have any influence on tumor and angiogenesis in a 3D model of tumor stroma interaction. The data provide a possible explanation for the failure of Cilengitide in phase III clinical trials by identifying the negative influence of endothelial cells on Cilengitide efficacy. In contrast, treatment of lung carcinoma cells with Cimetidine resulted in a striking reduction in tumor cell adhesion and transendothelial migration and treatment of different tumor entities in a 3D tumor stroma model provided evidence for an anti-tumor and anti-angiogenic effect of Cimetidine. Thus, the results suggest Cimetidine as a potential anticancer therapeutic that inhibits tumor cell adhesion and transendothelial migration through blockade of E-selectin and additionally inhibits tumor growth (potentially through the inhibition of H2 histamine) and angiogenesis.

## Data Availability

The original contributions presented in this study are included in the article/Supplementary Materials. Further inquiries can be directed to the corresponding author.

## Supplementary Materials

The following supporting information can be downloaded at: https://www.mdpi.com/article/10.3390/bioengineering13101192/s1, SM1. Materials and Methods. Figure S1. Overview of the 3D tumor–stroma co-culture model and experimental timeline. Figure S2. Response of HCT116GFP to treatment with 50 ng/ml Cilengitide and 200 µM Cimetidine on days 7 and 21. Figure S3. Adhesion assay with H838GFP on HPAEC endothelial cells treated with different concentrations of α4 antibody. Figure S4. Migration distance of H838GFP treated with Cilengitide (85 nM). Figure S5. Adhesion assay of HPEAC with different concentrations of Cilengitide.

## Abbreviations

The following abbreviations are used in this manuscript:

°C	degree centigrade
µg	microgram
µm	micrometer
1× PBS	single concentrated phosphate-buffered saline
3D	three dimensional
cm	centimeter
cR	exponent for constant kR
DMEM	Dulbecco’s modified eagle medium
EDTA	disodium salt dehydrate
FCS	fetal calve serum
g	gram
g	gravity in m/s^2^
GFP	green fluorescent protein
h	hour
H2-receptor	Histamin 2 receptor
H838	non-small lung carcinoma cells line
H838GFP	GFP transfected non-small lung carcinoma cells line
HCT116GFP	GFP transfected colon carcinoma cell line
HPAEC	human pulmonary arterial endothelial cell
HUVEC	human umbilical cord endothelial cell
m	meter
m	total mass
M	migration (M)).
mg	milligram
min	minute
mm	millimeter
PBS	phosphate-buffered saline
PC	polycarbonate
PDMS	polydimethysiloxane
PET	polyethylenterephtalate
PFA	paraformaldehyde
R	rolling (R),
RGD	cyclic arginyl-glycyl-aspartic acid
s	second
t	time
TEM	transendothelial migration
U87GFP	GFP transfected glioblastoma cell line
VE-Cadherin	vascular endothelial Cadherin
VEGF	vascular endothelial growth factor
