# Supplementary material for: Comparative Therapeutic Validation of a Microfluidic Metastasis Model: Cimetidine but Not Cilengitide Inhibits Physiological Tumor Cell Extravasation
Source: Bioengineering (Basel). 2026 Oct 14;13(10):1192. doi: 10.3390/bioengineering13101192 (PMC13701290; doi:10.3390/bioengineering13101192)
Supplement: Supplementary file 1 [file bioengineering-13-01192-s001.zip › bioengineering-4566412-supplementary.pdf]

Supplementary Materials for:

Comparative therapeutic validation of a microfluidic metastasis model: Cimetidine but not Cilengitide inhibits physiological tumor cell extravasation

Claudia Kühlbach[1,2], Lea Olschewski[2], Frank Baganz[1], Volker C. Hass[1,2], Margareta M. Mueller[2*]

[1] UCL University College London, Dept. Biochemical Engineering, London, UK; claudia.kuehlbach.17@ucl.ac.uk, f.baganz@ucl.ac.uk

[2] HFU Hochschule Furtwangen, Dept. Health, Medical and Life Sciences, Villingen-Schwenningen, Germany; claudia.kuehlbach@hs-furtwangen.de, margareta.mueller@hs-furtwangen.de, volker.hass@hs-furtwangen.de

* Corresponding author: margareta.mueller@hs.furtwangen.de

SM1. Materials and Methods

The vessel channel of the microfluidic device is approximately 500 µm × 100 µm and is lined with HPAECs, while a velocity of 8 mm/s is applied.

The corresponding wall interaction force has been calculated with the following formula:

$$F_{WI} = c_{WI} \cdot \rho \cdot \mu_A^2 \cdot \frac{a^6}{D_h^4}$$

With c_{WI}: wall interaction coefficient, ρ: density of medium, μ_A: sedimentation speed, a: area of cell, D_h: hydraulic diameter of vessel equivalent for other purposes and has not been used in this work. FWI = $8.319 \cdot 10^{-22(}$kg·m)/s^2.

To calculate the wall shear stress in the microfluidic channel, the following formula was executed: $T = 6\ \mu\ Q/\ (w\ h^2)$

with µ (fluid viscosity (Pa·s), Q (volumetric flow rate (m^3/s)), w (channel width (m)) and h (channel height (m))

➔ At a flow velocity of 8 mm/s: T= 4.617 dyn/cm^2

The value of T = 4.6 dyn/ cm^2 is lower than the values reported by in vivo measurements in arteries, e.g. in the mesenterium of cats. There conduit arteries show shear stress rates of around 10 dyn/ cm^2 (Lipowsky, H. H., S. Usami, and S. Chien "In vivo measurements of hematocrit and apparent viscosity in the microvasculature of cat mesentery." Microvasc Res 19 (1980): 297-319). Others report a wall shear stress of between 19.0 and 60.8 dyn/ cm^2 for arterioles and between 0.76 and 7.6 dyn/cm^2 in the venous system (Maringanti, Ranganath, et al. "Contributions of wall stretch and shear stress to vascular regulation: Molecular mechanisms of homeostasis and expansion." Vascular Mechanobiology in Physiology and Disease. Cham: Springer International Publishing, 2021. 21-46.). As the microfluidic device used in this manuscript has no musculature, it can be assumed that the value of the wall shear stress in the microfluidic device used here rather compares to the values, reported in venous blood system. Thus, with T = 4.6 dyn/ cm^2 the device lies within a physiological range.

S1. Experimental Overview

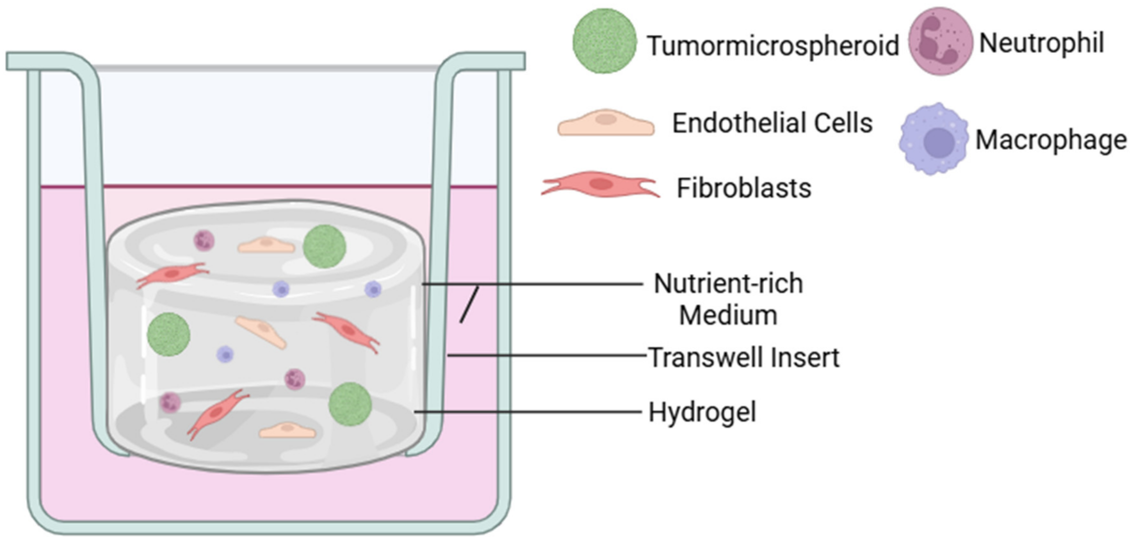

Figure S1. Overview of the 3D tumor–stroma co-culture model and experimental timeline.

3D-Tumor-Stroma models were generated using three different tumor cell entities (HCT116GFP, U87GFP and H838GFP) in combination with macrophages (U937-derived), neutrophils (HL-60-derived), fibroblasts (MSU1.1), and human primary endothelial cells (HUVEC). Cultures were treated with 50 ng/ml Cilengitide or 200 µM Cimetidine and monitored over 21 days. Fluorescence microscopy images were acquired on days 7, 10, 14, and 21. Created with BioRender.com

S2. 3D-Tumor-Stroma Model HCT116GFP

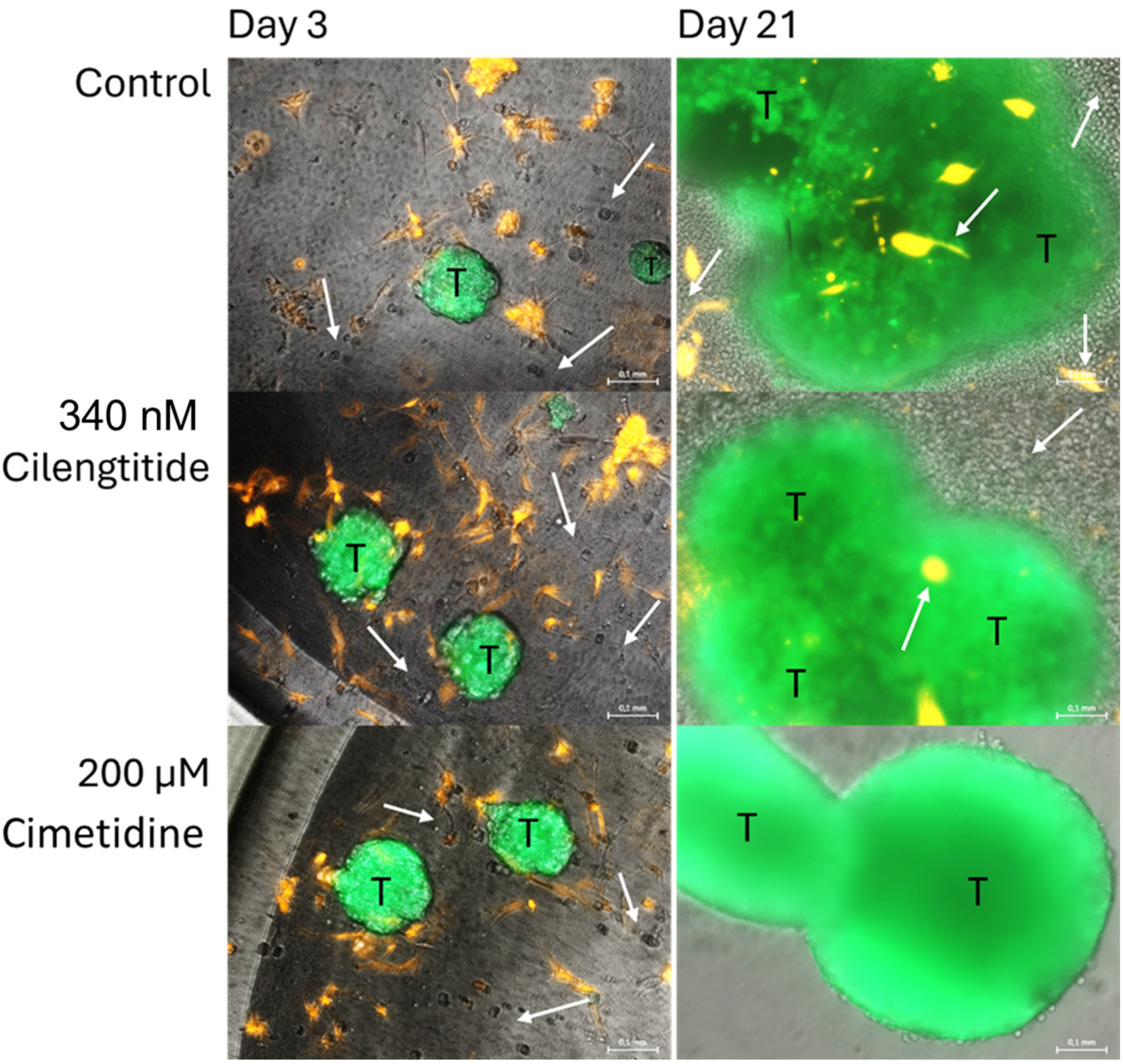

Figure S2. Response of HCT116GFP to treatment with 50 ng/ml Cilengitide and 200 µM Cimetidine on days 7 and 21

Representative fluorescence microscopy images of 3D tumor–stroma model comprising of HCT116GFP tumor spheroids, macrophages, neutrophils, fibroblasts (RFP), and endothelial cells. Cultures were treated with 340 nM Cilengitide and 200 µM Cimetidine and monitored over 21 days. Images were acquired on day 3 and day 21; Control conditions are shown for comparison. Tumor spheroids are labeled in green (T); endothelial cells labelled in red and human fibroblasts, macrophages and neutrophils are indicated with white arrows. Cilengitide did not show any effect on tumor spheroid growth nor on the cells in the stromal compartment. Treatment with Cimetidine revealed an inhibitory effect on the development of a tumor supporting stroma. Scale bars: [0,1 mm].

S3. Adhesion Assay with α4 antibody

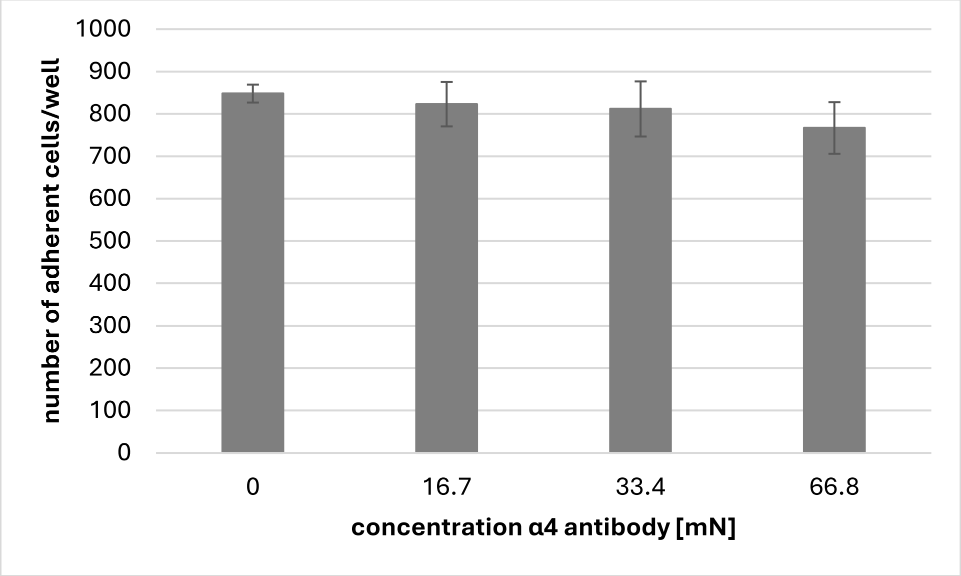

Figure S3. Adhesion assay with H838GFP on HPAEC endothelial cells treated with different concentrations of α4 antibody

The treatment with different concentrations of the α4 antibody shows no influence on the adhesion of H838GFP cells on HPAEC endothelial cells.

S4. Scratch Assay

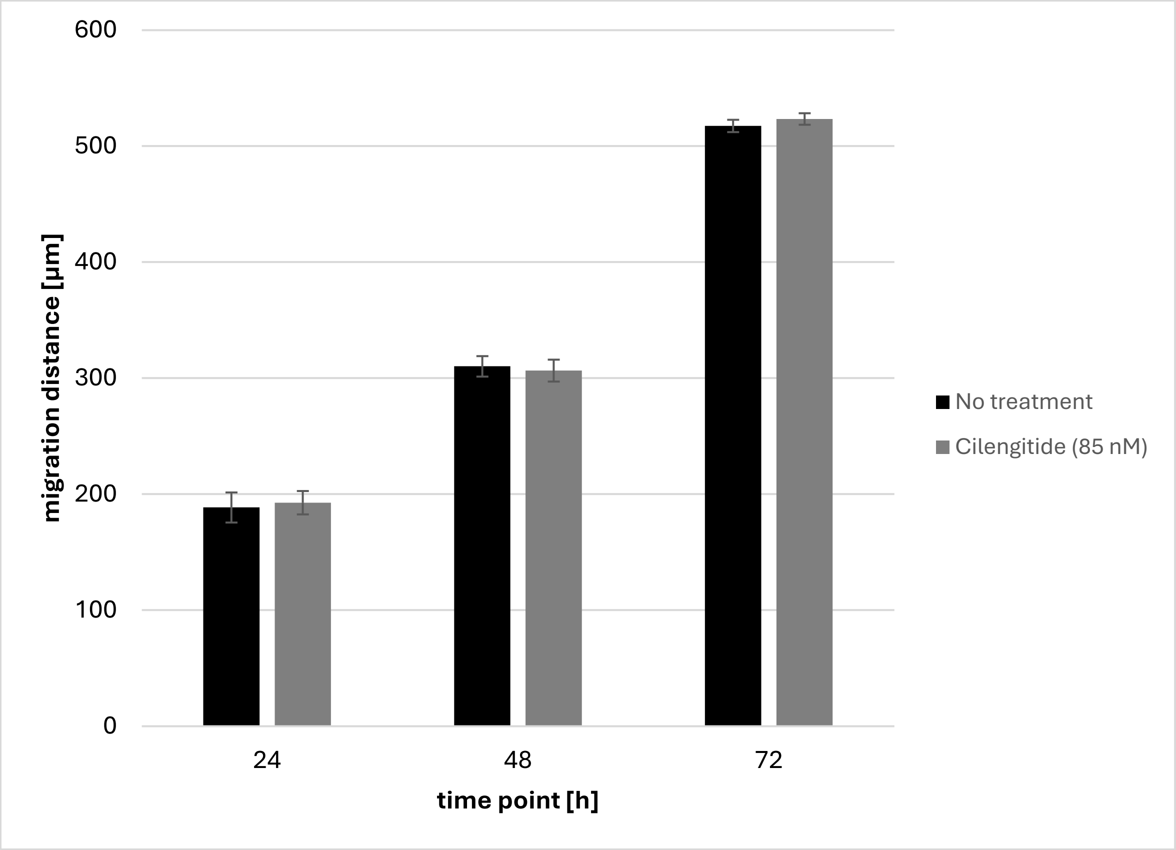

Figure S4. Migration distance of H838GFP treated with Cilengitide (85 nM)

The migration distance of H838GFP shows no changes when treated with 85 nM Cilengitide, compared to no treatment (0 nM) over the time of 72 hours.

S5. Adhesion Assay of HPAEC, treated with different

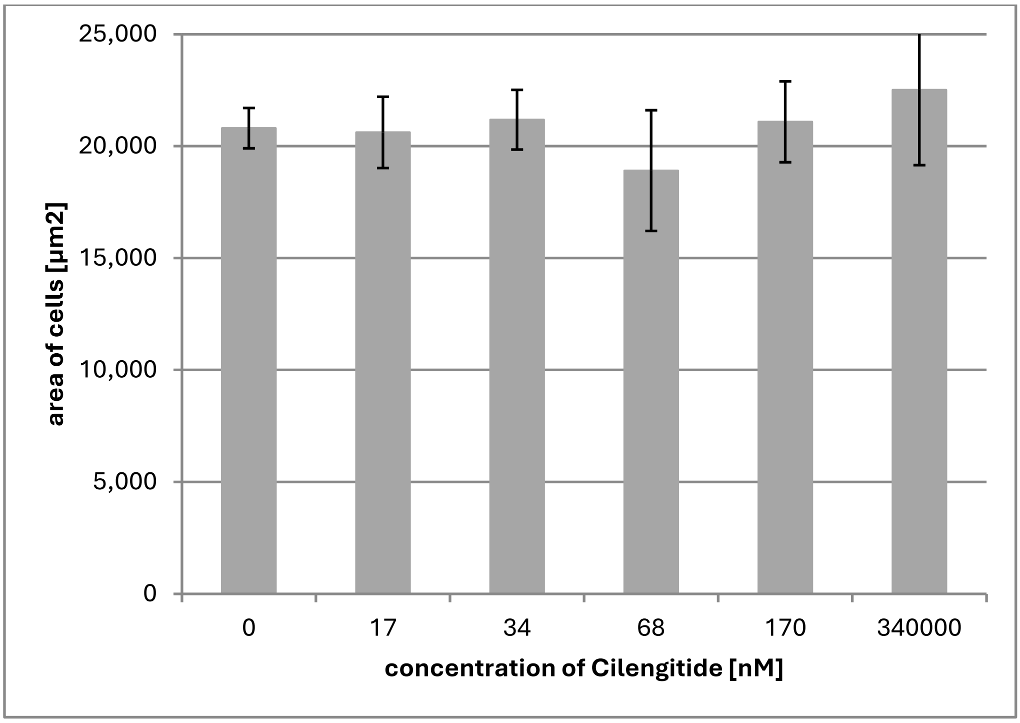

Figure S5. Adhesion assay of HPEAC with different concentrations of Cilengitide

The treatment of HPAEC endothelial cells with different concentrations of Cilengitide shows no influence, compared to no treatment.
